# Congenital Heart Defects in Patients with Anorectal Malformations: A Retrospective Cohort Study of 281 Patients

**DOI:** 10.1007/s00246-024-03536-3

**Published:** 2024-06-05

**Authors:** Cunera M. C. de Beaufort, Tara M. Mackay, Markus F. Stevens, Jorinde A. W. Polderman, Justin R. de Jong, Annelies E. van der Hulst, Bart Straver, Ramon R. Gorter

**Affiliations:** 1 Department of Pediatric Surgery, Emma Children’s Hospital Amsterdam UMC, Location University of Amsterdam, Meibergdreef 9, 1105 AZ Amsterdam, The Netherlands; 2 Amsterdam Gastroenterology and Metabolism Research Institute, Amsterdam, The Netherlands; 3 Amsterdam Reproduction and Development Research Institute, Amsterdam, The Netherlands; 4 Department of Surgery, Amsterdam UMC, Location University of Amsterdam, Meibergdreef 9, Amsterdam, The Netherlands; 5 Department of Anesthesiology, Amsterdam UMC, Location University of Amsterdam, Meibergdreef 9, Amsterdam, The Netherlands; 6 Department of Pediatric Cardiology, Emma Children’s Hospital Amsterdam UMC, Location University of Amsterdam, Meibergdreef 9, Amsterdam, The Netherlands

**Keywords:** Anorectal malformations, Children, Congenital heart defects, Echocardiography, Screening

## Abstract

In patients born with anorectal malformations (ARM), additional congenital heart defects (CHD) can occur. We aimed to provide an overview on disease and treatment details of CHD identified in patients born with ARM, from a unique large cohort of a very rare disease. We performed a retrospective single-center cohort study between January 2000 and July 2023. All consecutive patients with ARM were included. Outcomes were the number of patients with CHD, and screening percentage and percentage of patients diagnosed with CHD over 3 time periods (2000–2006, 2007–2014, 2015–2023). We used uni- and multi-variable logistic regression analyses to search for associations between CHD present and baseline characteristics. In total, 281 patients were included. Some 241 (85.8%) underwent echocardiography, of whom 80 (33.2%) had CHD. Screening percentage with echocardiography increased (74.1% vs. 85.7% vs. 95.9%, p < 0.001) and percentage of patients diagnosed with CHD remained similar over time (30.2% vs. 34.5% vs. 34.0%, p = 0.836). Atrial and ventricular septal defects (*n* = 36, *n* = 29), and persistent left superior vena cava (*n* = 17) were most identified. The presence of VACTERL-association or a genetic syndrome was independently associated with the presence of CHD. CHD were present in 33% of patients with ARM that underwent echocardiography. Over time, the number of CHD identified through screening remained similar. Patients with the presence of VACTERL-association or a genetic syndrome had a higher risk of having CHD. Therefore, acknowledging the potential presence of CHD in patients with ARM remains important.

## Introduction

Anorectal malformations (ARM) are rare colorectal disorders in which the anus is misplaced outside the external sphincter complex. This congenital disorder occurs in 1 to 3 in 5000 children each year [1]. In patients with ARM, additional congenital heart defects (CHD) can occur. For this reason, in most centers, all patients with ARM are routinely screened for CHD through physical examination (PE) and echocardiography within the first week of life [2]. The presence of CHD in patients with ARM is often part of (non-)syndromic anomalies such as Townes-Brocks and Down syndrome, or the VACTERL-association [3–5]. However, the overall incidence of CHD in patients with ARM differs widely as a range from 9 to 40% is described [6, 7]. Some patients with CHD require cardiothoracic surgery soon after diagnosis, whereas in others treatment can be somewhat delayed, or no surgery is needed at all [8, 9]. All patients with an ARM require surgical intervention in early childhood [10]. In order to prevent anesthesiological hazards due to missed CHD in these patients, it is essential to preoperatively identify those patients. Furthermore, regarding timing of surgeries for both ARM and CHD, it is helpful to know whether ARM patients have additional CHD.

However, to our knowledge, current literature lacks an accurate overview on how often and what kind of different CHD might occur in patients born with ARM. Therefore, the primary goal was to give an overview on the number of patients born with ARM and CHD, and the type of CHD that were identified. In order to provide optimal insight into the completeness of the presented cohort, the secondary aim of this study was to identify the number of patients that underwent postnatal echocardiography to identify CHD, with the screening tendency over time, the applied treatment for CHD in ARM patients (pharmaceutical, catheter intervention, and/or cardiothoracic surgery), and to identify factors associated with CHD.

## Material and Methods

### Study Design and Patient Population

At the Emma Children’s hospital from the Amsterdam University Medical Center (Amsterdam UMC) a prospective database consisting of all children born with an ARM since January 2000 until current is maintained. From this database patients were retrospectively selected for this current study which was set up in line with the STROBE guidelines (Strengthening the Reporting of Observational Studies in Epidemiology) [11]. For this study, all ARM patients born and/or treated in Amsterdam UMC from January 2000 until July 2023 were eligible for inclusion. In case parents objected to the use of data or when patients were born with an anus anterior (i.e., fistula located ≥ 50.0% within the external sphincter complex), they were excluded. Follow-up was determined as time between date of birth and date of latest clinical or outpatient clinic visit.

### Ethics

The medical ethical commission from Amsterdam UMC evaluated this project and decided that it was not amenable to the WMO statement (ref. no. W19_293 #19.350). Written information including a letter of objection was sent to patients, parents and/or legal guardians (in case of patients ≤ 11 years of age, only to parents and/or legal guardians; patients aged 12 to 15 years, both patient and parents and/or legal guardians; patients ≥ 16 years, patient only), of whom 6 objected to participate.

### Data Extraction

One of the authors (CB) extracted all data from the database on the 7th of August 2023. Validation of the extracted data was done by checking all cases with CHD by another author (BS and AH). The following data was extracted from the medical records of the included patients: baseline characteristics (i.e., gestational age and sex), form of ARM, genetic syndromes, VACTERL-association, type of CHD, other additional anomalies, cardiac imaging studies (i.e., echocardiography), consultation from a pediatric cardiologist, and type of treatment for CHD (pharmaceutical, catheter intervention, and/or cardiothoracic surgical).

### Definitions

The type of ARM was determined for each patient using the Krickenbeck classification [12]. Classification of VACTERL-association was based on the EUROCAT guideline article by van de Putte et al., comprising 4 categories: “STRICT-VACTERL (i.e., ≥ 3 major anomalies (in different organ systems) without other anomalies outside of the VACTERL-association), VACTERL-LIKE (i.e., ≤ 3 major anomalies plus minor anomalies adding up to ≥ 3 anomalies), VACTERL-PLUS (i.e., patients who fulfilled the strict-VACTERL or the VACTERL-like group, with additional anomalies outside of the VACTERL-association), NO-VACTERL (< 3 anomalies)” [3]. Gestational age was subdivided into pre-term (≤ AD 37 + 0 weeks), full-term (AD 37 + 0 through 40 + 6 weeks), late-term (41 + 0 through 41 + 6 weeks) and post-term (≥ 42 + 0 weeks) according to The American College of Obstetrics and Gynecologists (ACOG) guideline [13]. Full cardiac screening comprised of PE and echocardiography. All available echocardiography reports were examined to determine whether CHD were present in each individual patient. No imaging or echocardiography was repeated for any patient for study purposes. CHD were classified based on echocardiography findings, and subdivided into normal variants (i.e., transition phase related and/or neonatal period (≤ 1 month after birth) or specific clinical features (e.g., sepsis)) and structural CHD [14]. When a ductus arteriosus (DA) or oval foramen (FO) was identified on primary echocardiography, but not with follow-up echocardiography, or no follow-up echocardiography was performed, these findings were classified as normal variants, and not as CHD. In our cohort, as persistent ductus arteriosus (PDA) at the age of 1 month can still be the result of prematurity/neonatal transition rather than be a ‘true’ CHD, those patients with an isolated PDA at the age of 1 month were excluded (*n* = 3). In case a persistent oval foramen (PFO) was still present on follow-up echocardiography, it was classified as structural CHD.

### Outcomes

In this study, the number of patients with ARM in whom CHD were diagnosed was set as the primary outcome. In addition, secondary outcomes were the specific types of CHD diagnosed, the number of echocardiography performed, the screening tendency over time, percentage of patients diagnosed with CHD over the 3 time periods, the applied treatment for ARM patients with CHD (pharmaceutical, catheter intervention, and/or cardiothoracic surgery), and factors associated with CHD.

### Statistical Analysis

Regarding baseline characteristics and outcomes, only descriptive statistics were used. Binary and categorical variables were reported as proportions and percentages. Continuous variables are displayed as mean with standard deviation (SD) or as median with interquartile range (IQR), where suitable. For the secondary outcome screening tendency over time, 3 time periods (2000–2006; 2007–2014; 2015–2023) were formed. Statistical significance for screening tendency was evaluated using the Chi-square for trend. Type of ARM, gestational term, sex, presence of syndromes, presence of VACTERL-association, and enterostomy present were assessed with uni-variable analysis to identify possible associations with the presence of CHD. Those variables with a p < 0.10 were subsequently put into a model using multi-variable logistic regression analysis with backward stepwise selection. We demonstrated these outcomes as odds ratio (OR) with 95% confidence interval (95% CI). In this current study, a p-value of < 0.05 was set as statistical significant. Additionally, the proportion of variation in the occurrence of CHD in patients with ARM explained by the model (i.e., explained variance) was shown by the adjusted R-squared. We described all missing or unknown data. Regarding missing/unavailable data from additional imaging studies, we decided to classify it as ‘not carried out’. We used IBM SPSS Statistics for Windows, Version 28 (IBM Corp., Armonk, N.Y., USA) for the statistical analysis in this study.

## Results

### Participants

In total, 281 patients were included (period 1: *n* = 85, period 2: *n* = 98, period 3: *n* = 98), of whom 135 were female (48.0%) and 146 male (52.0%). Median age at follow-up of 7.0 years (IQR 3.0–12.0). Median gestational age was 38 weeks and 6 days (IQR 37 + 0 weeks–40 + 2 weeks). Recto-perineal fistula was most prevalent (*n* = 118, 42.0%), followed by recto-vestibular (*n* = 59, 21.0%) and recto-urethral (*n* = 44, 15.7%) fistulae. Some 111 patients (39.5%) had a stoma placed, and 257 patients (91.5%) underwent reconstructive ARM surgery (of which anterior and posterior sagittal anorectoplasty (ASARP, PSARP) most often). Syndromes were diagnosed in 48 patients (17.1%), of which caudal regression syndrome (*n* = 9), Cat-Eye syndrome (*n* = 6), Townes-Brocks (*n* = 5), and Down syndrome (*n* = 5) were most often identified. In total, 59 patients (21.0%) had a form of VACTERL-association. Overall, 220 patients (78.3%) had any additional anomaly. During the study period, 12 patients passed away due to various reasons, (i.e., premature birth with inoperable hypoplastic left heart syndrome (*n* = 1), abdominal compartment syndrome (*n* = 1), bacterial meningitis (*n* = 1), pneumococcal sepsis (*n* = 2), and respiratory insufficiency (*n* = 7)). No causality could be demonstrated with the presence of ARM. Table 1 provides an overview of patient characteristics.

**Table 1:** Characteristics of 281 patients with ARM

*Sex*	
Male	135 (48.0%)
Female	146 (52.0%)
*Type of ARM*	
Recto-perineal fistula	118 (42.0%)
Recto-vestibular fistula	59 (21.0%)
Recto-urethral fistula	44 (15.7%)
Recto-vesical fistula	7 (2.5%)
Cloaca	14 (5.0%)
Imperforate anus without fistula	17 (6.0%)
Anal stenosis	7 (2.5%)
Rare/regional variants	10 (3.6%)
Unknown type of fistula	5 (1.7%)
*Gestational term*	
Preterm	52 (18.5%)
Term	148 (52.8%)
Late term	24 (8.5%)
Post-term	8 (2.8%)
Unknown	49 (17.4%)
*Syndrome*	48 (17.1%)
*VACTERL-association*	59 (21.0%)
*Additional anomaly*	
Single	83 (29.5%)
Multiple	137 (48.8%)
*Colostomy*	
Colostomy placed	111 (39.5%)
Median age at colostomy in days (IQR)	2.0 (1.0–3.0)
*Reconstructive surgery*	
Reconstructive surgery performed	257 (91.5%)
Median age at reconstructive surgery in months (IQR)	4.0 (3.0–6.0)
*Median age at follow-up in years (IQR)*	7.0 (13.0–21.0)
Mortality*	12 (4.3%)

*ARM* anorectal malformation; *IQR* interquartile range; *VACTERL* vertebral, anorectal, cardiac, trachea-esophageal, renal, and limb anomalies

^*^During the study period, 12 patients deceased at ages 1, 2, 3 days, 1, 3, 5, 7 months, and 2, 16 years due to various reasons

### CHD

Screening for CHD with echocardiography was performed in 241 patients (85.8%), leading to the identification of CHD in 80 patients (33.2%). Fifty-five patients (68.8%) had a simple CHD, whereas 25 patients (31.3%) had complex CHD. Over the 3 time periods, the screening percentage increased (74.1% vs. 85.7% vs. 95.9%, p < 0.001), but the identified percentages of patients with CHD remained similar (30.2% vs. 34.5% vs. 34.0%, *p* = 0.836). Median age at primary echocardiography was 1.0 day (IQR 1.0–7.5), whereas the median age at follow-up echocardiography was 1.0 month (IQR 1.0–5.0).

**Fig. 1:**
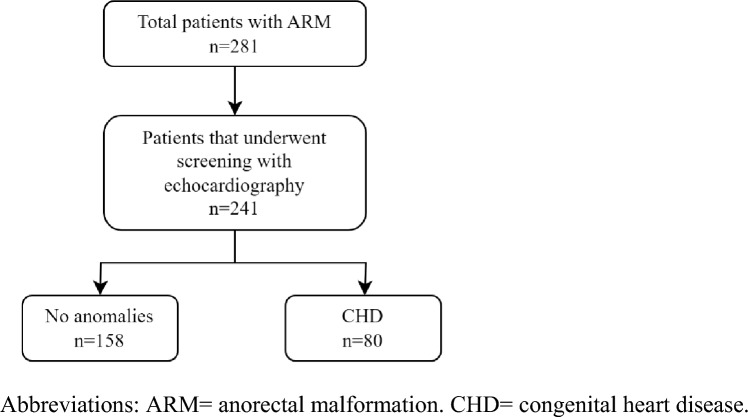
Type of CHD identified through echocardiography. ARM = anorectal malformation. CHD = congenital heart disease

Figure 1 depicts an overview of the number of patients in whom echocardiography was performed, with subsequent echocardiography findings. Overall, CHD were most often identified in patients with imperforate anus without fistula (8 of 15 patients (53.3%)) and recto-vestibular fistula (22 of 50 patients (44.0%)). Additionally, CHD were more often diagnosed in patients with intermediate complex types of ARM (simple 26.4% vs. intermediate 41.0% vs. complex 25.9%, *p* = 0.058). In total, 27 different types of CHD were identified. The most identified simple CHD were ASD (*n* = 36), VSD (*n* = 29), and persistent left superior vena cava (PLSVC) (*n* = 17). The most common complex CHD (*n* = 6) was Tetralogy of Fallot. An overview of the number of CHD identified through echocardiography per type of ARM is provided in Table 2. CHD were identified in both non-syndromic patients (*n* = 33) as well as patients in whom VACTERL-association and/or a genetic syndrome was present (*n* = 47), and the presence of a form of VACTERL-association (OR 4.04, 95% CI 2.14–7.65, p < 0.001) and a genetic syndrome (OR 2.94, 95% CI 1.43–6.05, *p* = 0.003) were independently associated with the presence of any CHD (see Table 3). This model has an explained variance of 14.5% (Nagelkerke R^2^ co-efficient).

**Table 2:** Type of CHD identified through echocardiography according to type of ARM

Type of ARM	Echocardiography performed	Cardiac anomalies
	n (%)	n (%)
Recto-perineal fistula, *n* = 118	103 (87.3)	28 (27.2)
Recto-vestibular fistula, *n* = 59	50 (84.7)	22 (44.0)
Recto-urethral fistula, *n* = 44	40 (90.9)	13 (32.5)
Recto-vesical fistula, *n* = 7	6 (85.7)	1 (16.7)
Cloaca, *n* = 14	13 (92.9)	5 (38.5)
Imperforate anus without fistula, *n* = 17	15 (88.2)	8 (53.3)
Anal stenosis, *n* = 7	3 (42.9)	0 (0.0)
Rare/regional variants, *n* = 10	8 (80.0)	1 (12.5)
Unknown type of fistula, *n* = 5	3 (60.0)	2 (66.7)
Total, *n* = 281	241 (85.8)	80 (33.2)

*CHD* congenital heart defect; *ARM* anorectal malformation; *n* number

^*^Percentages shown are calculated based on patients within the type of ARM that underwent screening with echocardiography

**Table 3:** Uni-and multi-variable analysis of the relation between baseline characteristics and the presence of CHD on echocardiography

	Univariable		Multivariable	
	OR (95% CI)	p-value	OR (95% CI)	p-value
*Sex*				
Female	Ref			
Male	0.71 (0.42–1.22)	0.219		
*ARM type*				
Simple	Ref			
Intermediate	**1.93 (1.08–3.46)**	**0.026**		
Complex	0.98 (0.37–2.55)	0.959		
*Gestational term*				
Term	Ref			
Preterm	1.38 (0.70–2.73)	0.351		
Late term	0.42 (0.14–1.33)	0.140		
Post-term	0.30 (0.04–2.56)	0.271		
*VACTERL-association*				
Not present	Ref			
Present	**3.81 (2.04–7.10)**	** < 0.001**	**4.04 (2.14–7.65)**	** < 0.001**
*Syndrome*				
Not present	Ref			
Present	**2.66 (1.33–5.31)**	**0.006**	**2.94 (1.43–6.05)**	**0.003**
*Stoma*				
Not present	Ref			
Present	1.38 (0.80–2.37)	0.249		

Bold in uni-variable analysis indicates variables (p < 0.10) that were entered in multi-variable analysis. Bold in multi-variable analysis indicates statistical significance (p < 0.05)

*ARM* anorectal malformation; *VACTERL* vertebral, anorectal, cardiac, trachea-esophageal, renal, and limb anomalies; *PE* physical examination; *OR* Odd’s ratio; *CI* confidence interval

### Treatment

A pediatric cardiologist was involved in all patients (100.0%) with CHD to define the appropriate treatment strategy. Thirty-one of 80 patients (38.8%) who had CHD required any form of treatment. In total, 20 patients (64.5%) required pharmacological treatment (i.e., diuretics, ibuprofen, or acetylsalicylic acid, 2 (6.3%) catheter intervention (i.e., Amplatzer device), and 25 (78.1%) cardiothoracic surgery for their CHD. In most patients, a combination of therapy was applicable. Median age at primary cardiothoracic surgery was 3.0 months (IQR 0.5–6.0). Two of 25 patients (8.0%) required multiple surgeries in order to resolve their CHD (Blalock-Taussig shunts in Tetralogy of Fallot). Over the 3 time periods, treatment percentages for CHD remained similar (12.9% vs. 10.2% vs. 10.2%, *p* = 0.798). A complete overview of types of CHD identified per type ARM is provided as Table 4.

**Table 4:** Type of CHD identified per type of ARM

Type of ARM	CHD	
	Type	n^#^
Recto-perineal fistula, n* = 28	Muscular VSD	11
	ASD type 2/PFO	5
	ASD type 2	5
	PLSVC	5
	Perimembranous VSD	2
	Coarctatio aortae with hypoplastic arch	2
	Coarctatio aortae	1
	Partial AVSD	1
	PDA	1
	Right aortic arch with a. lusoria	1
	Double aortic arch	1
	LVC with unroofed sinus coronarius	1
	Cor-triatriatum sinistra	1
	Tetralogy of Fallot	1
	Pulmonary artery sling	1
	Combined mitral valve anomaly	1
Recto-vestibular fistula, *n* = 24	ASD type 2/PFO	5
	PDA	5
	PLSVC	5
	ASD type 2	4
	Perimembranous VSD	3
	Muscular VSD	2
	Tetralogy of Fallot	2
	AVSD	1
	Coarctatio aortae	1
	Supracardial TAPVD	1
	Pulmonary artery sling	1
	Asymmetric tricuspid aortic valve with mild insufficiency	1
	Bicuspid aortic valve	1
Recto-urethral fistula, *n* = 13	ASD type 2	6
	Perimembranous VSD	4
	Aorta-pulmonary window	2
	Muscular VSD	2
	PLSVC	2
	Tetralogy of Fallot	2
	ARCAPA	1
	ASD type 2/PFO	1
	Coarctatio aortae	1
	Double orifice mitral valve	1
	PDA	1
	Right aortic arch with a. lusoria	1
	Unilateral absent pulmonary venous return	1
Recto-vesical fistula, *n* = 1	ASD type 2	1
	PLSVC	1
	Arteria lusoria	1
Cloaca, *n* = 5	ASD type 2	3
	PLSVC	2
	PDA	1
	Perimembranous VSD	1
	Tetralogy of Fallot	1
Imperforate anus without fistula, *n* = 9	ASD type 2	3
	Muscular VSD	2
	Perimembranous VSD	1
	ASD type 2/PFO	1
	AVSD	1
	PDA	1
	Right aortic arch with a. lusoria	1
	Arteria lusoria	1
	PLSVC	1
	Pulmonary artery sling	1
	Pulmonary valve stenosis	1
Rare/regional variants, *n* = 1	ASD type 2	1
	PDA	1
Unknown type of fistula, *n* = 2	Perimembranous VSD	1
	ASD type 2/PFO	1
	Coarctatio aortae	1
	PDA	1
	PLSVC	1
	Dextroposition	1
	Hypoplastic left heart syndrome	1
Total, *n* = 83		

*VSD* ventricular septal defect; *ASD* atrial septal defect; *PFO* patent foramen ovale; *AVSD* atrio-ventricular septal defect; *PDA* patent ductus arteriosus; *PLSVC* persistent left superior vena cava; *LVC* left vena cava; *TAPVD* Total anomalous pulmonary venous drainage; *ARCAPA* Anomalous origin of the right coronary artery originating from the pulmonary trunk

n* = number of patients in whom any CHD were diagnosed. n^#^ = number of CHD identified in patients

## Discussion

This retrospective cohort study provides an overview on all CHD identified in our cohort of patients born with ARM. Almost 34% of the patients with ARM that underwent echocardiography had CHD. In total, 27 different CHD were identified, of which ASD, VSD, and PLSVC most often. CHD were most often identified in patients with imperforate anus without fistula, but were also identified in more simple ARM types such as recto-perineal and –vestibular fistula. The presence of VACTERL-association or a genetic syndrome were independently associated with the presence of CHD. Furthermore, the majority of the patients with CHD required cardiothoracic surgery to resolve their CHD.

According to the findings of this study, CHD were present in almost 34% of the patients that underwent full cardiac screening. Compared to numbers reported in previous literature, this number is similar, but numbers of CHD in patients with ARM vary widely (9–40%) [6, 7, 15–17]. This might be caused by the wide range of anomalies classified under CHD in this study. However, echocardiographic findings such as PLSVC, right aortic arch (with a. lusoria), bicuspid aortic valve without stenosis or insufficiency or isolated arteria lusoria were classified as structural CHD, whereas other studies classify these anomalies as normal variant and not as CHD [18–21]. Classifying these anomalies as abnormal could potentially result in a higher number of CHD. Therefore, in our opinion, based on the number of patients with CHD identified in our cohort, treating physicians should acknowledge the potential presence of CHD in patients born with ARM.

Similar to previous studies, this study showed an association with the presence of VACTERL-association or a genetic syndrome with the presence of CHD in patients with ARM [22–24]. Patients with VACTERL-association were expected to have higher risk for the presence of CHD, since it is known that CHD are more often identified in these patients (because C in VACTERL covers CHD), also when ARM is not present [25]. Similar to VACTERL-association, patients with a genetic syndrome were expected to have higher risk for the presence of CHD [22]. In contrast to previous literature, in our cohort, pre-term patients with ARM did not seem to have a higher risk of CHD than at term patients with ARM (OR 1.38, *p* = 0.351) [26]. In addition, we tried to exclude PFOs that may still be present during neonatal from true ASDs by only including those when the pediatric cardiologist decided to perform follow-up after the age of one month. Moreover, future studies should investigate what the optimal timing should be to perform echocardiography as screening method for CHD in patients born with ARM, irrespective of type of ARM, and taking into account age at screening (e.g., premature birth vs. at term patients).

In our cohort, most children underwent screening through echocardiography early in life as the median age at echocardiography was 1 day. This median age at echocardiography included some outliers such as patients that were adopted or treated in Amsterdam UMC as second opinion, and therefore echocardiography was performed only at the age of 5–7 years. According to local hospital protocol, most patients underwent echocardiography primarily before stoma placement or reconstructive ARM surgery. Patients with a type of ARM requiring stoma placement early in life had a median age at stoma placement of 2 days. Moreover, since not all patients require a stoma placement within 48 h after birth, and some patients have CHD without any consequences for the anesthetist, it is questionable whether all patients with ARM should undergo echocardiography this early in life. However, we do not know how often an impact on operation planning and/or anesthesia was present that would not have been there if the child did not undergo screening for CHD. Additionally, based on the data available in this cohort, no hard conclusions can be made upon the timing for CHD screening, and within our center no changes were yet made in our daily practice. Therefore, it would be of great interest to perform future studies to evaluate the timing of cardiac ultrasound. Furthermore, diagnosing an ASD at the age of 1 year is not a problem from medical perspectives. However, early diagnosis might aid in understanding and parental coping. Hence, screening at a later moment in time might be justified, and it is therefore important to evaluate the optimal timing of screening.

As all studies with a retrospective character, results should be interpreted with care. The biggest strength of this study is that it provides one of the largest cohort describing patients with ARM in which CHD are identified over the past 23 years. Second, a large number of patients (86%) underwent cardiac screening with echocardiography, with an increasing trend over the 3 time periods (74% vs. 86% vs. 96%). No differences in detection rate were observed over the three screening periods. Accordingly, we may conclude that the numbers presented in this cohort are representative to the true prevalence or CHD in ARM. If any uncertainties regarding the classification of CHD occurred, a team of pediatric cardiologists and surgeons specialized in patients with ARM was consulted in order to evaluate the identified CHD in this cohort. The most important limitations of this study are selection and information bias due to the retrospective character and the relatively long study period. In order to reduce this to a minimum, consecutive data collection was performed, and data was checked by a second author (BS and/or AH). Additionally, if data on screening was not available in the medical record, it was classified as ‘not performed’. Because of the lacking of data, some (minor) CHD might not have been identified while they were present. In addition, given the fact that the cardiac ultrasound was performed early in life, and can therefore not yet differentiate between CHD or physiology, combined with the high prevalence of prematurity in this patient population, this remains a difficult discussion that needs more attention in future studies. Furthermore, cardiac screening can aid in the possibility to ensure well defined cardiac anatomy, and the primary neonatal team and the anesthesia teams can adjust if any precautions/management limitations might be needed. Moreover, it should be argued whether all forms of CHD can be viewed as “affecting management”, or if some (e.g., non-significant) CHD could be considered as not-affecting and/or influencing the timing of surgical treatment for ARM.

In conclusion, CHD were present in almost 34% of patients with ARM (regardless its severity) that underwent cardiac screening with echocardiography. Almost 39% of patients with CHD required any form of treatment at any moment in time. Presence of VACTERL-association or a genetic syndrome were independently associated with the presence of CHD. Therefore, acknowledging the potential presence of CHD in patients with ARM remains of great importance.

## Abbreviations

ARM: Anorectal malformations
CHD: Congenital heart defect
